# Will narrowing the educational gap between husband and wife alleviate housework inequality: Evidence from China

**DOI:** 10.3389/fpsyg.2022.1008210

**Published:** 2022-10-28

**Authors:** Zijian Peng, Lin Wu

**Affiliations:** School of Sociology, Wuhan University, Wuhan, China

**Keywords:** educational gap, housework inequality, instrumental variable method, income disparity, working time gap

## Abstract

In the past 20 years, China’s educational advantage has undergone a gender reversal. The average educational level of women is higher than that of men. However, the gender difference in housework is gradually expanding, and women are still the main undertakers of housework. Based on the China Family Panel Studies, this study explores the impact of the educational gap between husband and wife on the inequality of housework division and its mechanism. OLS regression model was used to estimate the impact of marital education gap on household inequality. It is concluded that the higher the education level of the husband is than that of the wife, the greater the gender inequality in housework. This conclusion is significant at the level of 0.01. On this basis, the instrumental variable method was used to overcome the endogenous problems and a more accurate conclusion was reached. Every unit of increase in the education gap between husband and wife would increase the degree of household inequality by 0.281 percentage points. Quantile regression provides strong evidence for the results. When the gender time ratio of housework is in the range of 0.8–0.95, the education gap will have an impact on the gender division of housework. After the robustness test and heterogeneity analysis of the model, an intermediary variable was established to discuss the mechanism of the model. The income disparity and the working time gap were proved to be intermediary variables. This study believes that in modern society, the education gap between husband and wife will affect the inequality of housework division by changing the relative income and relative working time of husband and wife. Although the educational advantages of women in the whole society have not changed their role in the division of housework. However, with the narrowing of the educational gap between husband and wife, the degree of inequality in the division of housework has been alleviated, indicating that the improvement of women’s education level has alleviated the inequality in the division of housework to a certain extent.

## Introduction

In recent years, gender reversal has occurred in China’s education, the average educational level of women began to be higher than that of men. According to the data of Statistical yearbook of China, the number of female college students in China exceeded that of male college students for the first time in 2009. The proportion of female college students reached 50.48% (National Bureau of Statistics of China, 2013). At the same time, the number of female master students in China exceeded that of male master students for the first time in 2010. The advantages of women in the field of education have increased year by year (Li, 2016). This means that the number of highly educated women who have reached marriageable age and are looking for a partner is beginning to exceed that of men. The change of educational background has directly changed the traditional marriage and mating mode, and people gradually tend to choose marriage partners with similar educational backgrounds (Grow and Bavel, 2015; Hauw et al., 2017). However, it is noteworthy that the impact of gender reversal in education on marriage matching at the social level has not affected the division of housework, and the traditional division of housework still exists. In the family, women are still the main undertakers of housework, and they spend about twice as much time on housework as men (Kornrich et al., 2013; Hu, 2015). According to the time use survey data of the National Bureau of statistics of China, in the 10 years from 2008 to 2017, although the housework time of both men and women in China has been shortened, the gender difference in housework time is gradually expanding. Compared with 2008, the housework time of men decreased by 32% in 2017, while that of women decreased by 27%. The housework time ratio of women to men increased from 2.91 in 2008 to 3.07 in 2017 (Fenglian et al., 2018).

As a classic topic, gender inequality in housework has been widely concerned by scholars for a long time. Under the background of the rising of global women’s power and the changing status, most of the studies on the division of household chores use the samples and perspectives of developed countries, which can not summarize and represent the situation of developing countries. Different from western countries, the gender awareness and behavior norms in developing countries are more traditional. The pattern of male dominating the outside and female dominating the inside is deeply rooted. Women in developing countries need to make more efforts than women in developed countries to obtain equal rights in marriage (Coltrane, 2000). Previous research on the gender inequality in housework in developing countries focused on income and gender awareness. First, for developing countries with underdeveloped economies, economic income is an important indicator to measure the status of families and individuals. The study points out that women’s absolute economic dependence on men forces them to assume the main family responsibilities, which is a supplement to men’s productive labor (Brenner, 1984; Gupta, 2006). Carrim (2017) found that when the economic contribution of the wife in the family gradually increased, they would ask for more equal division of labor with the husband after interviewing 25 double working Indian couples. Pinto and Coltrane (2009) established a prediction model of housework time for 393 Mexican families and found that when women get more income from the couple’s income, they will have greater bargaining power in the family and the housework time will be reduced accordingly. Second, although changes in the labor market can increase the bargaining power of women in developing countries in family affairs, it is difficult to change the gender division of housework due to the influence of traditional gender consciousness. Bittman et al. (2003) proposed in his research that gender inequality in family power caused by income inequality between men and women is based on gender neutrality, but this situation cannot completely cover all situations. In many developing countries, the division of housework depends more on the traditional sense of gender roles. Ahmed and Carrim (2016) pointed out that in India, which is deeply influenced by the traditional gender consciousness culture, women must obtain support from their husbands if they want to develop in work, and they must also undertake household responsibilities while developing work. Luke et al. (2014) conducted an interview with tea plantation workers in India, who are mainly female laborers, and found that when the wife’s income is higher than that of the husband, the husband will only help the wife to complete neutral family tasks such as cooking, collecting firewood and looking after children. Obviously, if we continue to use the previous interpretation of the division of housework, it is easy to conclude that the rise of female power in modern society makes women no longer the main bearers of housework. However, this conclusion is not consistent with the actual situation of Chinese society.

This study believes that previous studies have neglected the changes in marital education matching (relative education) brought about by the reversal of education gender in modern society. Some studies focus on individual education (absolute education), that is, the impact of women’s education as independent individuals on their marriage market, family structure and marital relations (Gonalons-Pons and Schwartz, 2017). Few studies have focused on the impact of the education gap between husband and wife (relative education) on the division of housework. As a relative resource, the educational gap between husband and wife determines their family power status and plays a major role in the division of family affairs (Blood and Wolfe, 1960; Tianshan and Qiaomin, 2021). In order to understand the gender inequality of housework division in the family under the background of gender reversal of education, this study takes relative education as the starting point to analyze whether the education gap between husband and wife can affect the inequality of housework division, and the impact path. This study uses the China Family Panel Studies (CFPS2018), and uses the family code to match the husband and wife data to test the impact of the education gap between each couple on the unequal division of housework. Further, quantile regression was used to study the impact of marital education gap on the unequal division of housework at different quantiles, revealing that there is not a simple linear relationship between the two. This paper also uses the instrumental variable method to verify the stability of the model on the basis of overcoming the endogenous problem, and analyzes the heterogeneity of the study from three dimensions: whether to use the Internet, age and marital satisfaction. On this basis, this study constructs a parallel intermediary model to explain the mechanism of the relationship between the educational gap between husband and wife and the unequal division of housework. It provides a more comprehensive perspective for us to understand the impact of the educational gap between husband and wife on the unequal division of housework.

## Literature review and research hypothesis

### Gender division of housework from the perspective of family power

The division of housework in the family is a process of establishing family gender order, which can be interpreted from the perspective of family power. The theory of domestic power is called the key to understanding family problems (McDonald, 1977). It not only directly reflects the mode of interaction between husband and wife, but also reflects the role division and family status of both spouses (Fuwa, 2004). From the root point of view, the establishment of family power is a game of husband and wife resources. In the study of family power, Blood and Wolf’s family power and authority model in 1960 is the most famous. The model points out that the relative resources of husband and wife determine their family power status, and the spouse with major resource advantages (such as education, income, occupation) will have more decision-making power, which is more likely to win in the daily power game between husband and wife. From the perspective of the realization of family functions, the distribution of resources in different gender roles will lead to a professional gender division of labor in the family, that is, by relying on each other’s advantageous resources, both husband and wife can make their marriage relationship more stable and get the maximum return (Becker, 1992). Schoen and Wooldredge (1989) research on the family points out that in general, in order to maximize the effectiveness of the family and stabilize the daily order of the family, husbands with resource advantages will exchange their wives’ domestic services by providing their own socio-economic resources. Therefore, from the perspective of family power, the resource disadvantage of wives compared with husbands is the main reason why they undertake housework in the family. The reasons for this gender division of family affairs have always been the focus of scholars’ research. After studying the division of housework and spouse resources in Britain, Sullivan and Gershuny (2016) found that family power comes from the comparison of resources reflecting the socio-economic status of couples, and people with more resources have more power to reduce housework time. The social and economic resources of individuals are always closely related to their education, which also makes education as a factor to measure the potential resource ability of individuals attract much attention (Schwartz and Han, 2014). With the promotion of industrialization and modernization, individual self-factors such as education have become the key factors of marriage matching and family relations. Blossfeld’s and Drobnic (2003) research points out that the unequal division of housework caused by women’s resource disadvantage compared with men in the family is due to their lower education level compared with their husbands. Kalmijn (2011) also proposed that men always dominate in the decision-making of housework division because their education level is higher than that of women. In the family, men have a higher level of education, and their potential to have advantageous resources is greater than women. Therefore, men control family power and occupy a dominant position in the division of housework.

Although with the development of economy and the popularization of higher education, women’s education level continues to improve, and the labor participation rate has increased significantly, there is a reversal of women’s education at the social level. However, it cannot be ignored that at the family level, the wife’s education level is still lower than that of her husband. This is because influenced by the traditional division of gender roles, men tend to choose women with lower social status, while women prefer men with higher education and professional class (Piotrowski et al., 2015). When the husband is in a weak position compared with the wife in marriage, it is difficult to be recognized by the mainstream family concept, and they will be considered to lack the sufficient ability to become husbands and fathers (Yu and Xie, 2015). So men usually don’t choose a wife with higher education than themselves. However, in the era of increasing cost of living, only relying on the economic income of men can not fully support the daily expenses of the family. Men will increasingly evaluate whether women are likely to become potential spouses according to their education and socio-economic status, and hope that women can share the economic and life pressure of the family with them (Oppenheimer et al., 1997).

To sum up, from the traditional gender perspective, the wife lacks the resource base to “negotiate” with her husband and is in a weak position in the family. To sum up, from the traditional gender perspective, the wife lacks the resource base to “negotiate” with her husband and is in a weak position in the family. The wife usually obtains “female resources” by doing more housework in exchange for her husband’s “male resources” and sharing her husband’s higher resource status (Jiping, 2002). However, with the progress of social development, women’s status has been improved through education, and the traditional marriage matching model has changed. In the family, the wife’s economic dependence on her husband has been significantly reduced, and they began to share the family economic pressure with their husband (Schwartz and Han, 2014). It can be seen that the “matching” of educational qualifications has become a more and more important condition for both men and women to choose a spouse. This also means that when both men and women are “balanced in strength” in marriage, their status division in family power will become more and more equal, and the gender differences in bearing economic pressure and domestic pressure will gradually narrow. Therefore, we propose the following hypothesis:

Hypothesis 1: The smaller the education gap between husband and wife, the smaller the status gap between them, and the more equal the division of housework in the family.

### Research on the mechanism of the influence of the educational gap between husband and wife on the division of housework

The industrialization theory proposes that the modernization and industrialization process make the degree of education significantly related to social and economic status. Rational people will choose marriage objects with higher social and economic status than themselves, so as to maximize their self-worth (Zijdeman and Maas, 2010). As the three elements of social and economic resources, income, work and education have become the key to determine the bargaining power of family members in the division of housework (Fuqin and Yuyin, 2020). Family members with status advantages often use higher bargaining power to avoid housework, and the result is that family members with lower status undertake most or even all of the housework (Perrucci et al., 1978). At present, the explanatory logic about the occurrence of this phenomenon is mainly concentrated in the time accessibility theory and the relative resource theory:

First, time accessibility theory: According to the time accessibility theory, the main concern of husband and wife in the family is the allocation of housework time and work time, and the maximization of the overall utility of the family can be achieved through the optimal time allocation cooperation. Beth (1992) pointed out in the research that the time spent by both husband and wife in housework should correspond to the time they spend in the labor market. Time constraints can largely explain the differences of family members in housework. As time and energy are limited, housework is generally undertaken by family members with relatively abundant time. For example, the wife’s housework time will decrease with the increase of their working hours, and the husband will correspondingly extend the housework time in order to cooperate with his wife (Kalleberg and Rosenfeld, 1990). The irreplaceable nature of time also determines that when family members divide family affairs, they should not only consider whether they are abundant, but also consider the value and cost of time. Therefore, how to devote limited time to things that can obtain greater value has also become an important basis for the division of housework. For example, after marriage and becoming a mother, women tend to invest more time in unpaid housework because of their mother’s nature, while men will invest more time in paid work because of their comparative advantage in income (Coverman, 1985). Hiller (1984) pointed out that the working time of family members are highly correlated with housework time, and the two show a negative correlation. Jia’s (2014) research found that the shorter the working hours of the husband and wife, the more flexible the work arrangement, and the longer the housework time. The fact that women undertake most housework is interpreted as their working hours are shorter than men. Jianlin (2017) proposed that in the family, both husband and wife should decide the division of housework according to their own resource advantages based on the principle of maximizing family interests. Family members with abundant time and low return on working time tend to bear more housework.

Combined with the relevant inference of human capital theory, the difference in working time investment between husband and wife can be explained by the difference in education level between husband and wife. The comparison of education level among family members may affect the input value of working hours through the difference of time value or opportunity cost in the labor market, and affect the housework time of both husband and wife. At the same time, the improvement of education level increases the economic cost of individual withdrawal from the labor market, which also increases people’s confidence and motivation to continue to invest in working hours, so it will reduce the time spent in housework. Higher education level is an important factor in the increase of labor supply behavior of married women. Some studies have pointed out that if the wife’s education level is higher than that of her husband, their investment in working time will be far greater than that of the wife whose education level is lower than that of her husband (Jie and Yafei, 2019). To sum up, the time accessibility theory provides a reasonable way for couples to balance family and work, and education is the key to improving the value of people’s working time. Based on the principle of maximizing family utility, family members with higher economic potential brought by higher education will spend more time in work and less time in housework. Therefore, we propose the following hypothesis:

Hypothesis 2: The educational level gap between husband and wife will lead to a gap in their working hours, which will further affect housework inequality: that is, the higher the education level, the more time they will invest in work, and the less time they will invest in domestic work.

Second, the relative resource theory: The relative resource theory puts forward that the division of family affairs reflects the power relationship between husband and wife to a certain extent, which is the result of the invisible game between husband and wife on their own resources (Blair and Lichter, 1991). Family members with more resources will have more power, and they are more likely to reduce their unwilling to participate in family activities, including housework and child care (Perrucci et al., 1978). The relative economic status of the spouse is an important factor to measure the amount of resources, which will affect the power relationship between the husband and wife. The spouse with a higher income will have a stronger ability to support their subjective choice in the distribution of family affairs, and the stronger the negotiation status of housework division, the more they can choose not to do or less housework (Pollak, 1996). Bittman et al. (2003) proposed that the spouse who controls more economic resources has a stronger negotiating position in family affairs, which can better achieve the expected results. Therefore, under the same conditions, having more economic resources in the spouse means less housework. Many empirical studies have proved the above view. Coverman’s (1985) research points out that when the husband has more income than his wife, these incomes will continue to strengthen their value in the labor market work, and the husband will spend less time in housework. Evertsson and Nermo (2004) also found that when the wife’s income is higher than the husband’s, the market work value is higher, and the wife’s housework input time will be reduced, and this reduction will be accompanied by the husband’s increase in housework time. Chinese scholar Liangshu (2005) pointed out, that income, as an important indicator of economic status in the family, determines the division of housework between husband and wife. The higher the income of a husband and wife, the more economic resources they occupy, the greater the power they have, and the more likely they are to have an advantage in bargaining about the division of housework. The main factor causing the income difference between husband and wife is the level of education. Education, as an important human resource, has a direct impact on individual income. The accumulation of human capital brought by the improvement of education and the optimistic expectation of future wages have increased the bargaining power of individuals in the division of housework. In recent years, the expansion of higher education in China has led to the expansion of academic qualifications, which has increased the supply of high-quality labor and directly increased the requirements of jobs for academic qualifications. From 2003 to 2008, the number of jobs requiring a university degree or above in China has increased sharply, which makes individuals with lower education have to accept lower wages and even withdraw from the labor market (Hu, 2013). It can be seen that the increase in income brought by the improvement of individual education will reduce their economic dependence on their spouse, and the decrease in their economic dependence on their spouse will reduce their housework time. The higher the income, the lower the economic dependence on the spouse and the shorter the housework time (Evertsson and Nermo, 2004; Kan, 2008; Sullivan and Gershuny, 2016). Therefore, we propose the following hypothesis:

Hypothesis 3: The educational level gap between husband and wife will lead to a gap in their working hours, which will further affect housework inequality: that is, the higher the education level, the more time they will invest in work, and the less time they will invest in domestic work.

**FIGURE 1:**
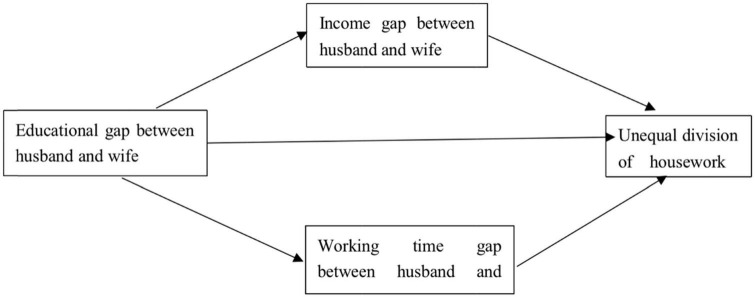
Research conceptual model.

Based on the above discussion, this paper believes that in the Chinese context, the educational gap between husband and wife will have an impact on the unequal division of housework. There are mainly the following two influence paths: There are mainly the following two influence paths: the first is that the difference in education between husband and wife makes their working hours different. Family members with shorter working hours have lower work intensity and have weaker family power, so they have to pay more housework. The second is that the difference in education level between husband and wife makes their income different. Family members with relatively low income have weaker family power, so they have to pay more housework. To sum up, we have established a conceptual model as shown in Figure 1.

## Data and methods

### Data sources

This study uses China Family Panel Studies (CFPS2018), which is a large-scale national social survey project carried out by Institute of Social Science Survey (ISSS). CFPS aims to reflect the changes of China’s society, economy, population, education and health by tracking and collecting data at three levels: individual, family and community. It is a national, large-scale and multidisciplinary social tracking survey project. The baseline survey was carried out in 2010, and the tracking survey was carried out every 2 years thereafter. Taking into account the regional differences in Chinese society, in order to save survey costs and improve the representativeness and scientificity of sample sampling, CFPS adopts a multi-stage, implicit stratified sampling method (PPS) proportional to the size of the population. CFPS2018 has five types of questionnaires: family members questionnaire, family economic questionnaire, individual self-administered questionnaire, children’s parents’ proxy questionnaire and individual proxy questionnaire, covering all family members in families and sample households in 25 provinces, cities and autonomous regions in China. The total sample size is 12,421 families and 32,669 individuals. The research object of this paper is the husband and wife in the family. In order to create a suitable database, we processed the data of CFPS as follows according to the research needs: First, the missing values, singular values and interruption samples are excluded, and the marriage status options are divorce, cohabitation, unmarried, widowhood and so on. Only the married option is retained, and a mixed sample of 2,186 women and 3,260 men is obtained. Secondly, according to the family number of the respondents in the family economic questionnaire, couples were matched for individuals with the same marriage year, forming a three-dimensional database of both husband and wife. After the above steps, 1,933 couples were finally obtained.

### Variable setting

**FIGURE 2:**
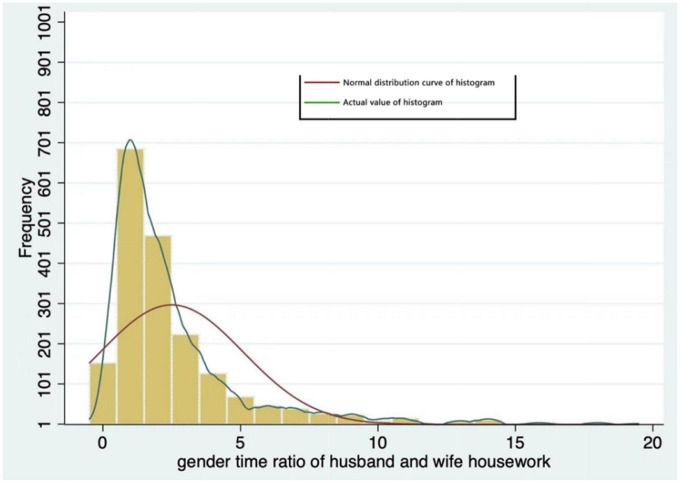
Distribution histogram of gender difference in housework division.

The explanatory variable of this study is “unequal division of housework.” Referring to the method of measuring gender differences in labor in the 2017 global gender gap report of the world economic forum, this variable is manipulated into the ratio of the total weekly housework hours of the wife to the total weekly housework hours of the husband (gender time ratio of husband and wife housework) (Fenglian et al., 2018). Different from the gender time gap, the gender time ratio excludes the influence of the base number. When the ratio is equal to 1, there is no difference in the division of housework between husband and wife, and when it is greater than or less than 1, there is a difference in the division of housework between husband and wife. The questions about housework time in CFPS2018 are divided into rest days and working days. This study will focus on personal housework time on working days × 5 + working and housework time on rest days × 2 get the total weekly housework hours of the individual. According to the calculation method of gender differences in work, we obtained a value from 0 to 20 after dividing the total weekly housework hours of men and women. The higher the value, the higher the degree of inequality in the division of housework between husband and wife. Based on this, the histogram of husband and wife’s housework division time ratio as shown in Figure 2 is drawn. According to the information in the figure, although the husband and wife’s housework division time ratio tilts to the left, most values are distributed at positions greater than 1, which shows that there are large gender differences in the housework division of Chinese families, gender inequality in housework is relatively common, and women are the main undertakers of housework.

The explanatory variable of this study is the “educational matching” of husband and wife. By integrating the questionnaire of CFPS2018, the husband and wife data are matched uniformly, and the education matching data of each husband and wife is obtained. This paper uses the method of measuring the educational level gap between husband and wife by Bing and Lige (2018) to code the individual’s educational level from illiterate/semi illiterate to master’s degree and above as 1–7, respectively, and subtract the wife’s educational level from the husband’s educational level to obtain the difference between the husband’s and wife’s educational level. In addition, we define that if the educational difference between husband and wife is greater than 0, that is, the husband’s educational level is higher than that of his wife, which is defined as “upward marriage.” If the educational difference between husband and wife is equal to 0, it is defined as educational “same marriage.” If the educational difference between husband and wife is less than 0, it is defined as education “downward marriage.” In order to better explore the impact mechanism of gender division of housework, this study also set up two intermediary variables: the income gap between husband and wife; the working time gap between husband and wife. On the basis of matching husband and wife variables, the wife’s annual income logarithm and weekly working hours are subtracted from the husband’s annual income logarithm and weekly working hours, respectively. Finally, we get two continuous variables: the income gap between husband and wife; the working time gap between husband and wife. In addition, in real society, there are many potential factors that will affect the inequality of housework in the family. Only after these factors are effectively controlled, can we effectively measure the impact of the difference in marital education on the inequality of housework division of labor. Therefore, we constructed a series of control variables, including the influence of respondents’ personal characteristics and family characteristics on the inequality of housework division, to test the independent effect of marital education matching characteristics. Specifically, the control variables include the respondents’ self-rated health, self-rated loneliness, working hours of working days, number of family housing units, family population, logarithm of family income, family housing property rights, and family consumption structure. At the same time, in order to eliminate regional differences, we also control the regional dummy variables. In order to overcome the possible endogenous problem of explaining the unequal division of housework with the difference of marital education, we set the instrumental variable “the average education gap between husband and wife in the same village/residence” as the instrumental variable.

### Model and analysis strategy

Consistent with previous studies (Xu and Yunhan, 2021), this paper uses ordinary least squares (OLS) to analyze the impact of marital education and marriage status on gender inequality in the division of housework between husband and wife in China, and designs the following regression equation:


(1) $$Y_{i}\,\alpha_{0}+\alpha_{1}\,X_{i}+\theta \,T_{i}+\epsilon_{i}$$

In Eq. 1, *Y_i_* represents the gender inequality in the division of housework between husband and wife in the ith family, which is a constant term, a control variable and a random error term, and *X_i_* represents the ith variable (*i* = 1, 2, 3,…, n) that affects the gender inequality in the division of housework among individuals. For the endogenous problems that may appear in Eq. 1, that is, some potential missing variables will affect the relationship between the education gap between husband and wife and the unequal division of housework. Equation 1 cannot effectively control these factors, so the results in the model may be biased. In order to deal with this potential endogenous problem, we use the instrumental variable method to re estimate Eq. 1. Specifically, it is divided into the following two stages:

Phase I estimate:


(2) $$X\,\beta_{0}+\beta_{1}\,{\text{Z}}_{i}+\beta_{2}\,{\text{T}}_{i}+\eta_{i}$$

Phase II estimate:


(3) $$Y_{i}\,\alpha_{0}+\alpha_{1}\,e\,s\,t\,i\,m\,a\,t\,e\,X_{i}+\alpha_{2}\,{\text{T}}_{i}+\epsilon_{i}$$

*Z_i_* is the instrumental variable. In this study, we use “the average education gap between husband and wife in the same village/residence” as the instrumental variable of the education gap between husband and wife. “The average education gap between husband and wife in the same village/residence” refers to the average education level gap between husband and wife living in the same village committee/neighborhood committee. In recent years, with the solidification of Chinese stratum and the expansion of regional development differences, the change of education level in the same region will have a significant impact on the education level of residents in the village/residence. To some extent, the degree of education received by the respondents is highly related to the overall level of educational development in the region. In other words, there is a strong correlation between the educational level gap between husband and wife in each family and the average education gap between husband and wife in the same village/residence. However, the relationship between the average education gap between husband and wife in the same village/residence and the characteristics of each family is weak. At the same time, because there is always a certain amount of population mobility, the specific division of housework of individual families will not be completely affected by the average education gap between husband and wife in the same village/residence.

To sum up, in order to accurately estimate the impact of marital education and marriage on household inequality. The operations of this paper are as follows: First, we use OLS regression model to estimate, and also use instrumental variable method to overcome the endogenous problem of the model. Second, a series of robustness tests are carried out on the estimation results by changing the model, changing the explanatory variables and the explained variables. Third, this study carried out quantile regression on the sample to completely describe the impact of marital education gap on the inequality of housework division at each quantile. Fourth, the heterogeneity analysis is used to test the conditions under which the impact of marital education gap on housework inequality will be established. Fifthly, the parallel intermediary model is used to explore and analyze the intermediary mechanism through which the educational gap between husband and wife affects the change of housework inequality.

## Results

### Variable description statistics

The statistical description of the samples finally used for empirical analysis obtained in this study is shown in Table 1. The first part is the descriptive statistics of mixed samples, and the second part is the descriptive statistics of husband and wife matching samples. At the social level, that is, from the mixed sample, women account for 40% of the total sample. The average weekly housework time of women is 14.5 h, and that of men is 8.7 h. The average weekly housework time of women is about 6 h higher than that of men. The average education level of women is about 0.1 higher than that of men, and women have a higher education level than men. At the family level, after matching the couple samples, we found that education “same marriage” and “upward marriage” accounted for 31 and 41%, respectively, and education “downward marriage” accounted for only 27%. Although from the perspective of the whole society, women’s education level is catching up with men in an all-round way, when choosing marriage, women still tend to choose education “upward marriage.” Therefore, in the family, the wife’s education level is generally lower than that of her husband, which is also in line with the serious situation of female doctoral celibacy in China. In the family division of labor, although the wife received more education, it did not significantly reduce her housework time. No matter the level of education is high or low, the wife is always the main bearer of housework. The average ratio of the wife’s housework time to the husband’s housework time is 2.5, which is significantly greater than 1. It can be seen that the wife’s housework time is significantly greater than the husband’s housework time, and the degree of gender differences in housework in the family is still relatively high.

**TABLE 1:** Descriptive statistics of samples.

Variable name	Mean value	Standard deviation	Minimum value	Maximum value	Total
** Descriptive statistics of mixed samples**					
Female housework time	14.451	9.758	0	91	2,186
Male housework time	8.652	8.322	0	77	3,260
Female education level	3.400	1.363	1	7	2,186
Male education level	3.308	1.233	1	7	3,260
Female housework time	48.566	19.110	1	168	2,186
Male housework time	52.157	19.014	0	168	3,260
Female education level	11.459	0.690	10	13	2,186
Male education level	10.478	0.765	8	12	3,260
Female working hours	3.085	1.049	1	5	2,186
Male working hours	3.233	1.085	1	5	3,260
Female personal income logarithm	1.451	0.656	1	4	2,186
Male individual income logarithm	1.359	0.625	1	4	3,260
Do women have two or more houses	0.271	0.445	0	1	2,186
Do men have two or more houses	0.269	0.444	0	1	3,260
Female family population	4.062	1.694	1	15	2,186
Male family population	4.316	1.872	1	14	3,260
Female family income (logarithm)	11.459	0.690	10	13	2,186
Male family income (logarithm)	11.362	0.674	10	13	3,260
Female family housing property ownership	0.862	0.345	0	1	2,186
Male family housing property ownership	0.880	0.325	0	1	3,260
Female household consumption structure	0.479	0.563	0	15	2,186
Male household consumption structure	0.470	0.309	0	7	3,260
** Descriptive statistics of husband and wife matching samples**					
Gender time ratio of husband and wife housework	2.501	2.591	0	19	1,933
Wife’s housework division time ratio	2.503	2.594	0	19	964
Husband’s housework division time ratio	2.498	2.590	0	19	969
Education gap between husband and wife	0.112	1.097	−5	4	1,933
The education of husband is higher than wife	3	0	3	3	596
The education of husband and wife is the same	2	0	2	2	785
The education of husband is lower than wife	1	0	1	1	521
Working time gap between husband and wife	3.262	23.612	−112	99	1,933
The husband works more hours than wife	3	0	3	3	911
Husband and wife work the same time	2	0	2	2	333
The husband works less than wife	1	0	1	1	673
Personal income gap between husband and wife	0.464	0.862	−2.960	3.485	1,933
The husband’s income is higher than wife’s	3	0	3	3	1,395
Husband and wife have the same income	2	0	2	2	112
The husband’s income is lower than wife	1	0	1	1	426
self-rated health	3.131	1.017	1	5	1,933
self-rated loneliness	1.397	0.645	1	4	1,933
Whether the family has two or more houses	0.319	0.466	0	1	1,933
Family population	4.231	1.765	1	14	1,933
Household income (logarithm)	11.592	0.600	10	13	1,933
Family housing property rights	0.886	0.318	0	1	1,933
Household consumption structure	0.457	0.312	0	7	1,933

In the mixed sample, the average working hours of women are about 4 h less than that of men, and the income (logarithm) of women is about 1 lower than that of men. After marriage matching, the working time gap between the wife and the husband was shortened to 3 h, and the working time of the wife was lower than that of the husband, accounting for nearly 50%. The average income (logarithm) gap between wife and husband is 0.46, and the wife’s income is lower than the husband’s income accounting for 72%. In the mixed sample, the average self-rated health of men and women are 3.23 and 3.10, respectively, and the average self-rated loneliness is 1.45 and 1.36, respectively. It can be seen that after marriage, the self-rated health score of men decreased, while that of women increased. The scores of men’s self-rated loneliness decreased, while women’s increased. From the comparison between the mixed sample and the husband and wife matching sample, it can be found that after entering marriage, the probability of having two or more houses in the family has increased, from 26.9 and 27.1% before marriage to 31.9% after marriage for men and women. The average number of male and female families increased from 4.31 and 4.06 before marriage to 4.23 after marriage. The family income (logarithm) of men and women increased from 11.46 and 11.36 before marriage to 11.59 after marriage. Before marriage, the probability of men and women owning housing property rights was 88.0 and 86.2%, respectively, and after marriage, this probability increased to 88.6%. The consumption structure of men and women in their families before marriage, that is, development consumption accounted for 47 and 48% of the total consumption, respectively, and this proportion fell to 45.7% after marriage. In conclusion, compared with before marriage, the health level and loneliness of men after marriage have decreased, while the health level and loneliness of women have improved. After marriage, the number of housing units in the family where the individual lives has increased, the family income has increased, the probability of owning family housing property rights has increased, and the family consumption structure has been improved.

### Variable correlation matrix

Table 2 presents the correlation matrix of the variables used in this paper. The data results show that, on the whole, there is a significant positive correlation between the education gap between husband and wife and the gender time ratio of housework. The correlation coefficient between the two is 0.057 (*p* < 0.1). With the widening of the educational marriage difference between husband and wife, that is, the higher the education level of husband is than that of wife, the greater the gender time ratio of housework is, and the higher the inequality of their division of housework is. Specifically, in the samples we used, there is a significant positive correlation between the working time difference between husband and wife and the gender time ratio of housework, and the degree of correlation between the two is 0.092 (*p* < 0.05). With the widening of the working time gap between husband and wife, the gender inequality in the division of housework will become more serious. There is also a significant positive correlation between the income gap between husband and wife and the gender time ratio of housework. The correlation coefficient between the two is 0.065 (*p* < 0.05). When the income gap between husband and wife continues to widen, their inequality in the division of housework is also more serious. In addition, there is a significant negative correlation between the number of family population and the gender time ratio of housework, and the correlation coefficient between the two is −0.061 (*p* < 0.05), which indicates that when the size of family population gradually increases, the inequality of housework division will weaken.

**TABLE 2:** Descriptive statistics and correlations among all variables.

	(1)	(2)	(3)	(4)	(5)	(6)	(7)	(8)	(9)	(10)	(11)	(12)
(1) Hwdevided	1											
(2) Edu_diff	0.057*	1										
(3) Worktime_diff	0.092**	−0.079**	1									
(4) Income_diff	0.065**	0.120**	0.105**	1								
(5) self-rated health	–0.019	–0.009	0.009	0.007	1							
(6) Worktime	–0.007	–0.007	−0.098**	0.039	0.025	1						
(7) Self-rated loneliness	–0.005	0.019	–0.024	0.034	−0.132**	0.036	1					
(8) Nhouse	0.012	0.004	0.038	–0.037	0.011	–0.009	−0.050*	1				
(9) Sfamily	−0.061**	0.008	–0.033	0.008	0.095**	0.032	–0.022	−0.061**	1			
(10) Ln_fincome	0.002	–0.005	–0.034	−0.134**	0.023	−0.144**	−0.076**	0.002	–0.005	1		
(11) Hproperty	0.005	0.008	0.012	–0.011	0.016	–0.023	–0.036	0.005	0.008	0.012	1	
(12) Hcs	–0.031	–0.004	–0.022	–0.002	0.016	0.053*	–0.035	–0.031	–0.004	–0.022	–0.002	1

*p < 0.1; **p < 0.05.

### Ordinary least squares regression results

On the basis of correlation analysis of relevant variables, through OLS regression analysis, and using the method of gradually increasing control variables to observe the fitting degree of the model, analyze and test the correlation between the education gap between husband and wife in China and the unequal division of housework between husband and wife. The specific results are shown in Table 3. Model 1 shows that when the control variables are not included, and only the education gap between husband and wife and the gender time ratio of housework are included in the model, there is a significant positive correlation between the two, and the correlation coefficient is 0.135, which is significant at the level of 0.05. On the basis of model 1, model 2 takes the influencing factors of self-rated health, self-rated loneliness and working hours at the individual level as control variables into the model. After that, there is still a significant positive correlation between the education gap between husband and wife and the gender time ratio of housework. The correlation coefficient is 0.134, which is significant at the level of 0.05, and the fitting degree of the model has been significantly improved. On the basis of model 2, model 3 takes the influencing factors at the family level, whether the family has two or more houses, the number of family population, the logarithm of family income, whether it has family housing property rights, and the structure of family consumption (the proportion of family development consumption in total consumption) as control variables into the model, and the educational gap between husband and wife and the gender time ratio of housework are still positively correlated. The correlation between the two has been significantly improved, at the level of 0.01, and the fitting degree of the model has been significantly improved. Considering the potential regional effect of the location of the family, we add the regional dummy variables in model 4 in addition to the control variables included in model 3. The results show that the fitting degree of model 4 is the highest compared with the first three models. At this time, the educational gap between husband and wife still has a significant positive impact on the inequality of housework division, which is significant at the level of 0.01. After gradually adding control variables, we find that the fitting degree of the model is higher and higher, and the estimation results are more and more significant, which fully explains the rationality of the model selection. We propose that the education gap between husband and wife has a positive impact on the inequality of housework division, and support hypothesis 1.

**TABLE 3:** Ordinary least squares (OLS) regression results.

	Model 1	Model 2	Model 3	Model 4
Edu_diff	0.135** (2.51)	0.134** (2.49)	0.156*** (2.87)	0.158*** (2.90)
Self-rated health		−0.039 (−0.67)	−0.037 (−0.62)	−0.031 (−0.53)
Self-rated loneliness		0.020 (0.21)	0.013 (0.14)	0.019 (0.20)
Worktime		−0.002 (−0.70)	−0.002 (−0.51)	−0.002 (−0.47)
Nhouse			0.016 (0.12)	0.008 (0.06)
Sfamily			−0.070** (−1.96)	−0.066* (−1.83)
Ln_fincome			0.074 (0.70)	0.105 (0.96)
Hproperty			0.433** (2.31)	0.390** (2.07)
Hcs			−0.028 (−0.15)	−0.006 (−0.03)
Region	No	No	No	Yes
_cons	2.486*** (42.01)	2.693*** (9.40)	1.720 (1.34)	1.211 (0.93)
N	1,933	1,933	1,933	1,933
r^2^	0.003	0.004	0.009	0.013

*p < 0.1; **p < 0.05; ***p < 0.001.

### Endogenous problem

The unequal division of labor in housework may be related to many potential factors. Explaining the unequal division of labor in housework only by using the educational gap between husband and wife may produce large deviations. In order to solve this endogenous problem, were estimated the benchmark regression using the instrumental variable method. Table 4 presents the results of the re estimation of the model. Among them, (1) is the estimation result of the first stage of the model, (2) is the estimation result of the second stage of the model. After estimating the model, we tested whether the instrumental variable “the average education gap between husband and wife in the same village/residence” has the problem of weak instrumental variables. The results show that the joint significant statistic *F*-value of instrumental variables is far greater than 10 (*f* = 578.33, *p* < 0.001), which means that there is a strong correlation between instrumental variables and endogenous independent variables. At the same time, according to our previous theoretical presupposition, “the average education gap between husband and wife in the same village/residence” is difficult to establish a relationship with the division of housework between husband and wife. In this sense, we believe that “the average education gap between husband and wife in the same village/residence” is a very reasonable instrumental variable in this paper. The results in Table 4 well confirm this point. The first stage estimation results show that the instrumental variable “the average education gap between husband and wife in the same village/residence” has a significant positive impact on the endogenous independent variable “education gap between husband and wife,” and has no significant correlation with the dependent variable. Model 1, model 2, and model 3 are significant at the level of 0.001, respectively. At the same time, with the gradual increase of control variables, the goodness of fit of the model has improved. The results of the second stage show that after we use the instrumental variable method to re estimate the model, the educational gap between husband and wife still has a significant impact on the inequality of housework division in the family. Among them, model 4, model 5, and model 6 are significant at the level of 0.001, respectively. The estimation coefficient of the model is higher than that of the benchmark regression, and the goodness of fit of the model also increases with the increase of control variables. In contrast, the results of re estimation using instrumental variable method will be more accurate and reliable. For the variables controlled by the model, the estimation results are basically consistent with OLS regression results. It can be found from the results that the education gap between wife and husband has a significant positive impact on the inequality of housework division in the family. Specifically, every increase in the education gap between husband and wife will increase the degree of inequality in housework by 0.281 percentage points. Couples with low education gap are more likely to have a more equal division of housework. This reflects the educational gap between husband and wife, which is of great value to the distribution of housework chores.

**TABLE 4:** Estimation results of instrumental variable method.

	(1) Model 1	(1) Model 2	(1) Model 3	(2) Model 4	(2) Model 5	(2) Model 6
Medus	1.000*** (55.36)	0.999*** (54.47)	0.999*** (54.10)			
Edus				0.249*** (3.82)	0.277*** (4.28)	0.281*** (4.32)
_cons	0.030 (0.40)	−0.266 (−0.78)	−0.274 (−0.79)	2.692*** (9.19)	1.728 (1.30)	1.261 (0.96)
Control variable	Yes	Yes	Yes	Yes	Yes	Yes
Region	No	No	Yes	No	No	Yes
N	1,935	1,898	1,898	1,935	1,898	1,898
r^2^	0.614	0.611	0.612	0.002	0.007	0.011

***p < 0.001.

### Robustness check

Through the above analysis, we can know that there is a clear correlation between the education gap between husband and wife and the inequality of housework division. The more educated the husband is than the wife, the higher the inequality of housework division. In order to further test the robustness of the estimation results, and to have a more comprehensive and detailed understanding of the problems to be studied, we used three methods to test the robustness of the estimation results: replacing the model, replacing the explained variables, and replacing the explanatory variables. The specific results are shown in Table 5.

**TABLE 5:** Robustness test results.

	(1) Change model	(2) Change the interpreted variable	(3) Change explanatory variables
Edu_diff	0.162*** (2.97)	0.612*** (3.12)	0.275*** (3.55)
Self-rated health	−0.024 (−0.40)	−0.236 (−1.41)	−0.030 (−0.51)
Worktime	−0.002 (−0.47)	−0.007 (−0.63)	−0.002 (−0.46)
Self-rated loneliness	0.020 (0.22)	0.384 (1.37)	0.020 (0.22)
Nhouse	0.012 (0.09)	−0.767** (−2.34)	0.007 (0.05)
Sfamily	−0.067* (−1.87)	−0.220** (−2.43)	−0.068* (−1.89)
Ln_fincome	0.099 (0.90)	−0.977*** (−3.42)	0.107 (0.98)
Hproperty	0.386** (2.04)	0.148 (0.28)	0.373** (1.99)
Hcs	−0.010 (−0.05)	−0.023 (−0.06)	−0.000 (−0.00)
Region	Yes	Yes	Yes
_cons	1.268 (0.96)	(.) −0.467	0.645 (0.49)
N	1,898 (−0.94)	1,898	
r^2^	0.003	−0.390	0.016

*p < 0.1; **p < 0.05; ***p < 0.001.

First, as the explained variable, the gender time ratio of husband and wife housework is a continuous variable, so we choose OLS model as the benchmark regression model. Since the housework time is greater than 0, the gender time ratio of husband and wife housework is a positive number greater than or equal to 0, which is a very typical blocking data, and there is no negative situation. The characteristics of this data meet the requirements of Tobit model for data, so this paper uses Tobit model to test the research problem in addition to OLS regression. The data results in the first column of Table 5 show that after Tobit regression is used, there is still a positive correlation between the education gap between husband and wife and the gender ratio of housework time, which is significant at the level of 0.01, that is, the greater the education gap between husband and wife, the more serious the inequality of their housework division.

Second, the explained variable used in OLS regression is the “unequal division of housework.” We use the existing research for reference, and turn it into “the gender time ratio of husband and wife housework,” which excludes the influence of the base number. In order to interpret the inequality of housework in a more comprehensive and detailed way, we take into account the base number. By subtracting the wife’s housework time from the husband’s housework time, we get the time difference between husband and wife’s housework. After changing the explanatory variables, were estimated the model by using the instrumental variable method, and obtained the data in the second column in Table 5. Consistent with the above conclusion, the more educated the husband is than the wife, the higher the inequality of housework in the family. There is a positive correlation between the two, and it is significant at the level of 0.001.

Third, in this study, the education gap between husband and wife is operationalized as a continuous variable. However, at present, the educational gap between husband and wife is mostly regarded as a categorical variable in the academic circles, that is, the education level of the husband subtracting the education level of the wife is divided into three types: those greater than 0 are “upward marriage,” those equal to 0 are “same marriage,” and those less than 0 are “downward marriage.” Therefore, we replace explanatory variables with categorical variables. The results in the third column of Table 5 show that there is still a positive correlation between the education gap between husband and wife and their unequal division of household chores, which is significant at the level of 0.001. Consistent with the results of OLS regression, the probability of unequal housework time increases by 27.5% for every unit of increase in the education gap between husband and wife.

After changing the model, re operating the explanatory variables and the explained variables, the conclusion is still consistent with the OLS regression model. Therefore, from all the test results, the regression results of this paper are basically robust.

### Quantile regression

**FIGURE 3:**
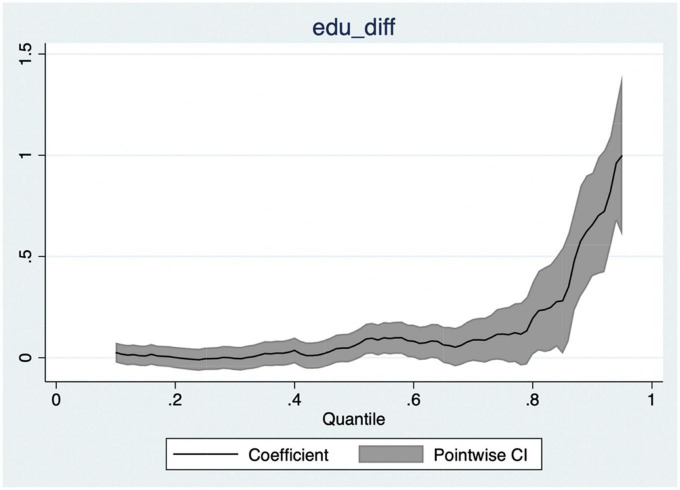
Quantile regression coefficient diagram.

In the OLS regression model, we mainly investigate the correlation between the education gap between husband and wife and the “average” inequality in the division of housework. However, OLS model cannot estimate how various factors change in the overall distribution of this variable. Koenker and Bassett proposed quantile regression, assuming that the conditional quantile of the explained variable is a linear function of the explained variable (Zhi et al., 2021). By constructing a quantile regression model, the change and influence of influencing factors on the distribution position of the explained variable are revealed, and the influence of the explained variable is better displayed on the overall distribution of the explained variable. In order to more comprehensively describe the relationship between the educational gap between husband and wife and the inequality of housework division, we used quantile regression model to test the impact of the educational gap between husband and wife on the gender time ratio of housework at different quantiles. Figure 3 shows the quantile regression coefficient diagram of the marital education gap and other control variables affecting the inequality of housework division. The quantile interval set in this study is 0.1–0.95, and the interval in the middle is 0.01. Figure 3 shows that the influence of the education gap between husband and wife and other control variables on the inequality of housework division is not non-linear. When the gender time ratio of husband and wife housework is below 0.8 quantile, the impact of changes in the marital education level gap on the domestic sex time ratio is below 0, and close to a straight line, which shows that in this range, no matter how the marital education level gap changes, the value of the gender time ratio of husband and wife housework will not change. In this quantile range, the effect of marital education gap on the gender time ratio of husband and wife housework is 0. When the gender time ratio of husband and wife housework is in the range of 0.8–0.95 quantiles, the marital education gap has a significant positive impact on the gender time ratio of husband and wife housework, and the impact effect increases with the increase of the quantile of the gender time ratio of husband and wife housework. It shows that when the inequality of housework is low, the education gap between husband and wife will not play a role, and the positive impact of the education gap between husband and wife on the inequality of housework will only be reflected in the high gender time ratio of husband and wife housework.

### Heterogeneity analysis

In order to investigate the conditions under which the education gap between husband and wife will affect the inequality of housework division, this study analyzed the heterogeneity of the samples. Table 6 presents the heterogeneity analysis results of the impact of marital education gap on the inequality of housework division. The results show that after controlling all the covariates controlled by the above benchmark regression model, when people do not use the Internet, the educational gap between them and their spouses will affect the unequal division of housework in the family. When people use the Internet, the educational gap with their spouses has no significant impact on the unequal division of housework in the family. This is because most groups who do not use the Internet are those who have more traditional ideas, and their family concepts are more traditional than those who use the Internet. Therefore, the educational gap with their spouses will have a significant impact on the division of housework of groups who do not use the Internet.

**TABLE 6:** Heterogeneity analysis results.

	(1) Use	(2) Not used	(3) 20–35 years old	(4) 36–50 years old	(5) 51–60 years old	(6) Dissatisfied	(7) General	(8) Satisfied
Edus	0.073 (0.74)	0.193*** (3.01)	0.345*** (3.08)	0.097 (1.37)	0.029 (0.21)	−0.199 (−0.72)	−0.042 (−0.25)	0.182*** (3.11)
_cons	1.577 (0.64)	1.582 (1.01)	6.873** (2.33)	0.573 (0.35)	−5.947* (−1.80)	−15.023* (−1.99)	8.567** (2.32)	0.565 (0.40)
Control variables	Yes	Yes	Yes	Yes	Yes	Yes	Yes	Yes
*N*	415	1,481	524	1,083	289	34	169	1,693
*r* ^2^	0.016	0.020	0.040	0.019	0.047	0.592	0.067	0.015

*p < 0.1; **p < 0.05; ***p < 0.001.

Relevant research shows that the largest difference in housework time between women and men occurs between the ages of 51–55, and the peak of housework time between women and men occurs between the ages of 26–30 and 31–35, respectively (Fenglian et al., 2018). Therefore, after comprehensive consideration, we divide the age of the sample into three stages: 20–35, 36–50, and 51–60 years old. The results in Table 6 show that when the age group is 20–35 years old, the education gap between people and their spouses will have a positive impact on the inequality of their housework division of labor. When the age group is 36–50 and 51–60 years old, the educational gap between husband and wife will not have an impact on their unequal division of housework. This also means that age has become a regulating factor between the educational difference between husband and wife and the inequality of housework division. When people’s age gradually increases, the educational difference between people and their spouses will no longer have an impact on the inequality of housework division. We also pay attention to the impact of different marriage satisfaction, and divide people’s evaluation of their marriage into three categories: dissatisfied, general and satisfied. According to the results in Table 6, when people are satisfied with their marriage, the education gap with their spouses will have a positive impact on the inequality of housework division in the family. When people evaluate their marriage as dissatisfied and average, the impact of education gap on the division of housework is no longer significant. This is also in line with the reality of China. Women generally have higher marital satisfaction after “climbing high,” which means that the husband’s relative resources are higher than his wife. At this time, the division of labor in the family tends to be “male dominated outside and female dominated inside.”

### Mechanism analysis

**FIGURE 4:**
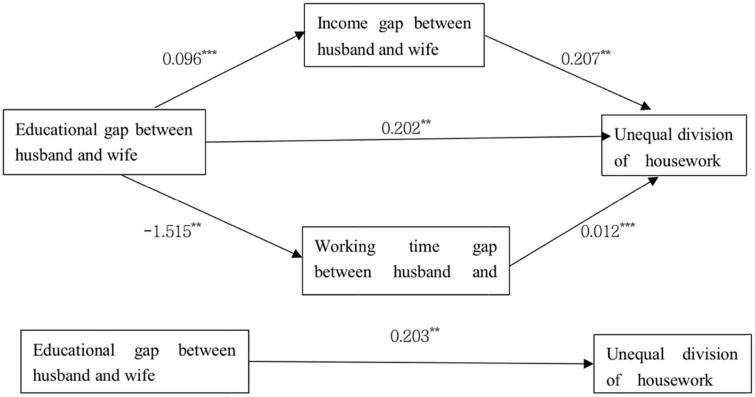
Test results of parallel mediation model. **p < 0.05; ***p < 0.001.

In order to better understand the relationship between the education gap between husband and wife and the inequality of housework division in China, we further explored and analyzed the potential intermediate mechanisms through which the difference in education level between husband and wife may affect the change in the inequality of housework. Specifically, this paper constructs a parallel mediation model: one is the educational gap between husband and wife–the working time gap between husband and wife–the unequal division of housework, the other is the educational gap between husband and wife–the income gap between husband and wife–the unequal division of housework, and uses the deviation correction percentile Bootstrap method to test the mediation effect. It can be seen from Table 7 and Figure 4 that the gap between husband and wife’s working hours and income are effectively supported by the data. The total indirect effect was also statistically significant. The total effect of marital education gap on household inequality is 0.203, and the confidence interval does not include 0, indicating that the total effect is significant. On the basis of controlling other variables, the direct effect of marital education gap on household inequality is 0.202, and the confidence interval does not include 0, so the direct effect is significant. In addition, the total mediation effect is 0.038, and the confidence interval does not include 0, indicating that the total mediation effect is significant. The path of the total intermediary effect can be decomposed into: the effect of the education gap between husband and wife on the income gap between husband and wife is 0.096, which is significant at the level of 0.001. The effect of the income gap between husband and wife on household inequality is 0.207, which is significant at the level of 0.05. And the Bootstrap 95% confidence interval of income gap does not include 0, which has a significant mediating effect. The effect of the educational level gap between husband and wife on the working hours gap between husband and wife is −1.515, which is significant at the level of 0.05. The effect of the working hours gap between husband and wife on household inequality is 0.012, which is significant at the level of 0.001. The Bootstrap 95% confidence interval of the gap between husband and wife’s working hours does not include 0, and the mediating effect is significant. Through the analysis of the parallel mediating effect, we can know that the two mediating effects exist. The educational level gap between husband and wife will affect the inequality of housework division in the family through their income gap and work hours gap. The income gap and working time gap between husband and wife are important influence mechanisms to explain the relationship between marital education gap and household inequality.

**TABLE 7:** Parallel mediation effect.

Various effects	Effect value	Boot standard error	Boot CI lower limit	Boot CI upper limit
Total effect	0.203	0.073	0.061	0.346
Direct effect	0.202	0.073	0.058	0.345
Total mediating effect	0.038	0.011	0.019	0.063
Working hours gap	–0.018	0.008	−0.037	–0.005
Income gap	0.020	0.008	0.006	0.036

To sum up, we propose that the education gap between husband and wife has a positive impact on the inequality of housework division through the working time gap between husband and wife, and support hypothesis 2. We propose that the education gap between husband and wife has a positive impact on the inequality of housework division through the income gap between husband and wife, and support hypothesis 3.

## Discussion

This paper believes that in China, the education gap between husband and wife is the key factor affecting the unequal division of housework. This paper provides some insights into the impact of individual absolute education and relative education on the division of housework. The improvement of absolute education can increase the competitiveness of individuals in the marriage market. Well educated women are becoming more and more popular in the marriage market. The economic potential brought by higher education has increasingly become an important standard for men to choose a spouse (Zhou et al., 2017). Relative education is the result of marriage education matching between husband and wife. It is an important indicator that affects the establishment of family power. It can not be ignored in the formation of husband wife relationship and the maintenance of marriage order (Jianlin, 2016). Under the influence of the patriarchal system in China for a long time, the concept of gender roles and housework are not independent. The dominant position of women in housework is the gender inequality in the family shaped by the interaction of the economic status of men and women in the labor market and the social and cultural expectations for different genders. Under the influence of the male dominated system in China for a long time, the concept of gender roles and domestic work are not independent. The dominant position of women in housework is the gender inequality in the family shaped by the interaction of the economic status of men and women in the labor market and the social and cultural expectations for different genders. This phenomenon has not changed qualitatively with the reversal of gender in China’s social education and the improvement of women’s educational status, which also proves that the improvement of individual absolute education can only improve their self-worth in the job and spouse selection market. The traditional marriage concept of “men are high and women are low” still continues in China’s educational marriage matching. According to previous studies, women’s economic dependence on their husbands is the main reason why they have to undertake housework, and the income gap between men and women is considered to be the key to women’s social subordination (Brenner, 1984). When women’s education level is high enough, it means that they have high economic potential, then the attraction of traditional marriage mode and marriage order to them will be reduced, and they may choose to invest more time and energy in work. This is considered to be contrary to the traditional female image role, which makes them face the problem of marriage difficulties (Qian and Sayer, 2015). Therefore, the situation that the wife has higher education than the husband does not occupy the mainstream. In the family, the husband is still the main dominator of resources and power.

However, we can not ignore that, with the narrowing of the educational gap between husband and wife, the inequality in the division of housework will indeed weaken, which also shows that the improvement of women’s education level will alleviate the inequality in the field of housework to a certain extent. Although the traditional gender division of labor cannot be broken in a short time, with the development of the times, the popularization of higher education has brought about the improvement of women’s power and the improvement of men’s quality, as well as the narrowing of the income gap between family members and the gap in the employment market, which will inevitably make the gender difference in household division of labor smaller and smaller. At the same time, people should realize that the construction of modern harmonious family relations is not based on the unequal division of labor of housework on the basis of weakening women’s status and sacrificing women’s interests. Instead, it redefines the nature of housework with innovative thinking, close to people’s real life style and the concept of gender equality, and promotes the harmonious relationship of mutual understanding and support among family members. Equal domestic relations are one of the important means to realize and promote mutual support and encouragement between husband and wife.

## Conclusion

This study uses the 2018 CFPS data to study the impact of the education gap between husband and wife on the unequal division of housework in China. Like western society, China has experienced a reversal of the gender gap in the field of education in the past two decades (Wu and Zhang, 2017), and the probability of similar marriage in the field of education has increased to a certain extent (Han, 2010). However, the family gender division model of “male dominated outside and female dominated inside” has not been completely changed, and the gender boundary of family affairs division is very obvious (Juhua, 2006). The reason and mechanism of this phenomenon are still not completely clear. As an important measure of social and economic status, the educational gap between husband and wife has an important impact on the division of family affairs. The greater the educational gap between husband and wife, the more unequal their division of housework. The reason and mechanism of this phenomenon are still not completely clear. As an important measure of social and economic status, the educational gap between husband and wife has an important impact on the division of family affairs. The greater the educational gap between husband and wife, the more unequal their division of housework. In China, under the marriage culture dominated by patriarchy, although women’s education level has overtaken that of men and widely entered the labor market, it has not changed the state of “men are higher than women” in educational marriage. According to our research, at the social level, the average educational level of women is higher than that of men, but after entering marriage, the proportion of wives with higher educational qualifications than husbands only accounts for 26.9%. Obviously, the influence of traditional gender concept and marriage matching on people’s mate selection has not disappeared with the gender reversal in the field of education. The all-round improvement of education level is also changing people’s gender and family concepts. The probability of marriage of the same kind of education with similar education level has greatly increased, accounting for 41%. However, it cannot be ignored that there are still a large number of educational gradient marriages (non-educational marriage of the same kind) in society because the influence of the traditional gender role division model has not disappeared in the short term. Therefore, in order to comprehensively consider the impact of the existing education matching on the existing marriage and family relationship, this study takes the marital education gap as a continuous variable to explore its relationship with household inequality. In the context of gender reversal in education at the social level and changes in marriage matching in education at the family level, this study explores the inequality in the division of housework and draws the following conclusions:

First, this study verifies the hypothesis that the greater the educational gap between husband and wife, the higher the degree of inequality in the division of housework. When the educational gap between husband and wife increases, it is proved that the resources of husband and wife are unequal in the family, and their power status also varies greatly, so the housework is more likely to be concentrated in family members with lower education. Second, based on OLS regression, this study uses instrumental variable method to overcome the possible endogenous problems of the model. The instrumental variable “the average education gap between husband and wife in the same village/residence” is set to retest the model, and it is found that the results are more accurate and reliable. This also proves that the education gap between husband and wife has important value for the unequal division of housework. Thirdly, this study conducted a robustness test on the basis of OLS regression model. The results were re estimated by replacing the model, replacing the explanatory variables and replacing the explained variables. The results showed that they were still consistent with the conclusions of OLS regression model. Fourth, considering that the degree of inequality in the division of housework is not a unified “average” level, in order to better investigate its overall distribution, this study adopts the method of quantile regression, and comes to the conclusion that the gap between husband and wife’s education level will have an impact on the inequality of housework only in the gender time ratio of housework with high scores. Fifthly, in order to investigate the conditions under which the education gap between husband and wife will affect the inequality of housework division, this study takes whether to use the Internet, age and marital satisfaction as the classification criteria, and uses the method of heterogeneity analysis to explore the relationship between the education gap between husband and wife and the inequality of housework division. Sixthly, by setting two intermediary variables, the income gap between husband and wife and the working hours gap between husband and wife, this study deeply discusses the mechanism path of the education gap between husband and wife in the unequal division of housework.

### Strengths and limitations

The main strengths of this study are as follows. First, understanding the impact of the educational gap between husband and wife (relative education) on the unequal division of housework is an important expansion of the existing literature on gender differences in education and housework division. Previous research perspectives mostly focused on the education level of individuals (absolute Education), and there was less research on the education gap between husband and wife (relative education), but the education gap between husband and wife and the education level of individuals are two different concepts. Second, from the perspective of the educational gap between husband and wife, this study discusses the mechanism of inequality in the division of housework, which enriches the existing forms of the impact of marriage education matching on the division of housework. Previous studies have mostly studied the educational matching of husband and wife as a categorical variable (Education upward marriage, education downward marriage, education homogeneous marriage), In order to comprehensively investigate the impact of the existing education matching on marriage and family relations, this study takes the education matching of marriage as a continuous variable, that is, to explore the relationship between the educational level difference between husband and wife from small to large and the inequality of housework. Third, by investigating the impact of the educational gap between husband and wife on household inequality, this study explains why the traditional gender division of household work is still stable under the background of the reversal of educational gender and women’s socio-economic status catching up with men. This study further emphasizes the important role of the difference in educational resources between husband and wife in the determination of family power and its impact on maintaining the gender order of the family, and also provides an explanatory idea for explaining the single phenomenon of a large number of highly educated women in today’s society.

This study also has the following limitations. First, due to the influence of many practical factors, this study only uses cross-sectional data, which can not dynamically reflect the relationship between the education gap between husband and wife and the unequal division of labor in housework. Further research should consider multi-year longitudinal data analysis and dynamically pay attention to the annual change of gender division of domestic work in China. Second, due to the adoption of cfps2018 questionnaire, the questionnaire does not reflect the issue of individual subjective consciousness. The division of housework is not only an objective phenomenon, but also inseparable from the subjective cognition of family members. This limits the study of individual self-motivation. In the future research, we should adopt diversified questionnaires to capture the role of individual subjective consciousness in the gender division of housework. Third, this study matched couples one by one, and eliminated many missing values in the process, resulting in a small sample size. In the future research, we should continue to expand the sample size to make the research results more representative and popularized.

## Data availability statement

The original contributions presented in this study are included in the article/supplementary material, further inquiries can be directed to the corresponding author.

## Ethics statement

Ethical review and approval was not required for the study on human participants in accordance with the local legislation and institutional requirements. Written informed consent from the patients/participants OR patients/participants legal guardian/next of kin was not required to participate in this study in accordance with the national legislation and the institutional requirements.

## Author contributions

ZP: conceptualization, methodology, software, data curation, writing–original draft preparation and review, and editing. LW: supervision, investigation, project administration, and funding acquisition. Both authors contributed to the article and approved the submitted version.
